# Supplementary material for: SARS-CoV-2 Within-Host and in vitro Genomic Variability and Sub-Genomic RNA Levels Indicate Differences in Viral Expression Between Clinical Cohorts and in vitro Culture
Source: Front Microbiol. 2022 May 19;13:824217. doi: 10.3389/fmicb.2022.824217 (PMC9161297; doi:10.3389/fmicb.2022.824217)
Supplement: Supplementary file 1 [file Table_1.DOCX]

**Supplementary Table**

**Supplementary Table S1:** Median sgRPTL by gene and lineage.

|  | **Gene** | | | | | | | | |
| --- | --- | --- | --- | --- | --- | --- | --- | --- | --- |
| **Lineage** | **ORF1ab** | **S** | **ORF3a** | **M** | **ORF7a** | **ORF8** | **N** | **ORF6** | **E** |
| **B.1** | 5.474 | 12.452 | 95.553 | 22.549 | 42.748 | 13.674 | 60.246 | 0.397 | 0 |
| **B.1.617.2** | 0 | 2.987 | 13.617 | 15.383 | 11.957 | 5.262 | 39.946 | 0.386 | 0.311 |
| **B.6** | 0 | 7.848 | 32.987 | 33.102 | 20.912 | 6.126 | 0 | 0 | 0 |
| **D.2** | 0.273 | 38.501 | 56.811 | 86.419 | 84.859 | 39.981 | 139.286 | 7.173 | 7.335 |
