# Supplementary figures and images for: SARS-CoV-2 Within-Host and in vitro Genomic Variability and Sub-Genomic RNA Levels Indicate Differences in Viral Expression Between Clinical Cohorts and in vitro Culture
Source: Front Microbiol. 2022 May 19;13:824217. doi: 10.3389/fmicb.2022.824217 (PMC9161297; doi:10.3389/fmicb.2022.824217)

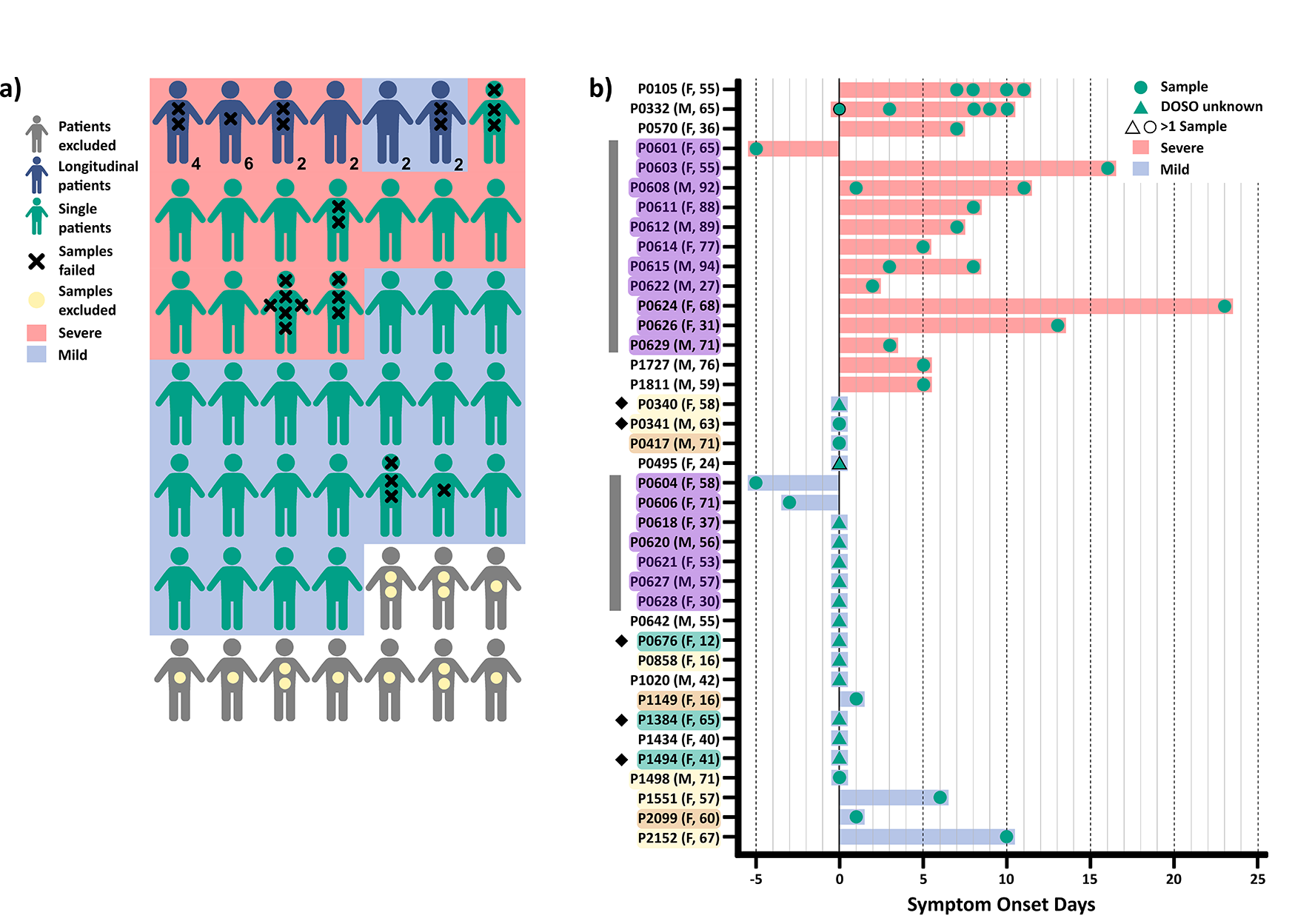

Supplement: Supplementary Figure 1 — COVID-19 cohorts and sampling timeline. (A) Representation of the initial patient pool considered for this study. Patients excluded (grey) and their respective samples (yellow circle) did not pass quality filtering. The pink and light blue blocks represent the severe and mild cohorts respectively. Within cohorts, the blue silhouettes represent longitudinally sampled patients and green represents patients where only one sample was taken. The number of longitudinal samples included in the study are indicated but the numerical values below the silhouette. The number of samples in the cohort that failed quality filtering are illustrated by the black cross. (B) The cohort type and duration of onset days of enrolled patients. Samples of patients with known symptom onset (green circle) and unknown symptom onset (green triangle) are illustrated. When more than one sample was taken on the same day, the shape is outlined in black. Severe and mild cases are shaded with red and blue, respectively. Patients infected with SARS-CoV-2 Delta lineage are represented by grey vertical bars. Patients within the same genomic epi-cluster are grouped by purple (Lineage B.1.617.2, cluster NSW 130), yellow (Lineage D.2, cluster NSW 33.1), orange (Lineage D.2, cluster NSW 33), and green (Lineage B.1, cluster NSW 17.5) shaded boxes. Black diamonds denote household contacts within the genomic epi-cluster. [file Image_1.TIFF]

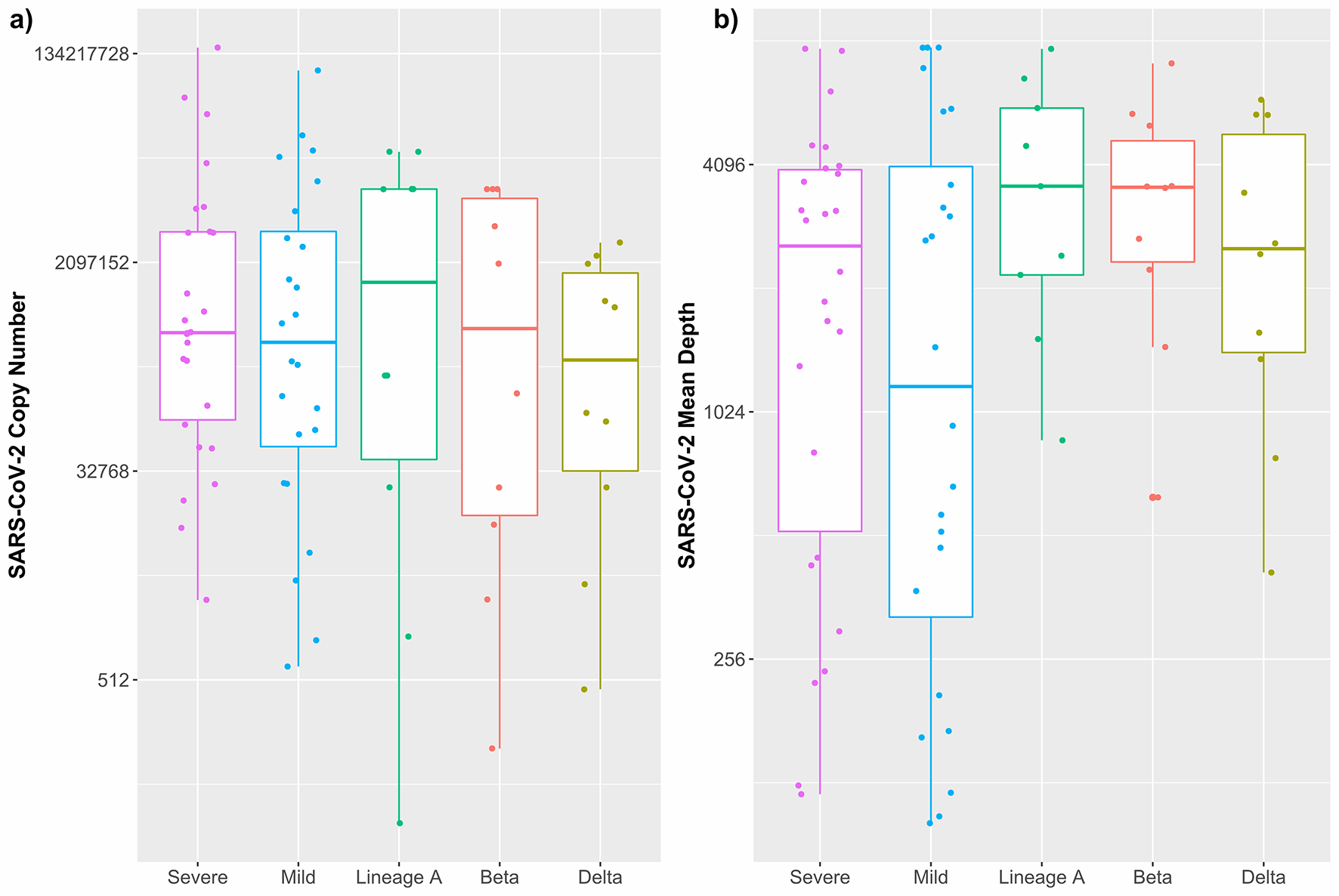

Supplement: Supplementary Figure 2 — Boxplots depicting the SARS-CoV-2 copy number (A) and median genome depth (B) for all cohorts. The bold line indicates the median, the interquartile range (25th to 75th percentile) is represented by the white shading, the whiskers represent the minimum and maximum values, and the outliers are shown by the black circles. [file Image_2.TIFF]

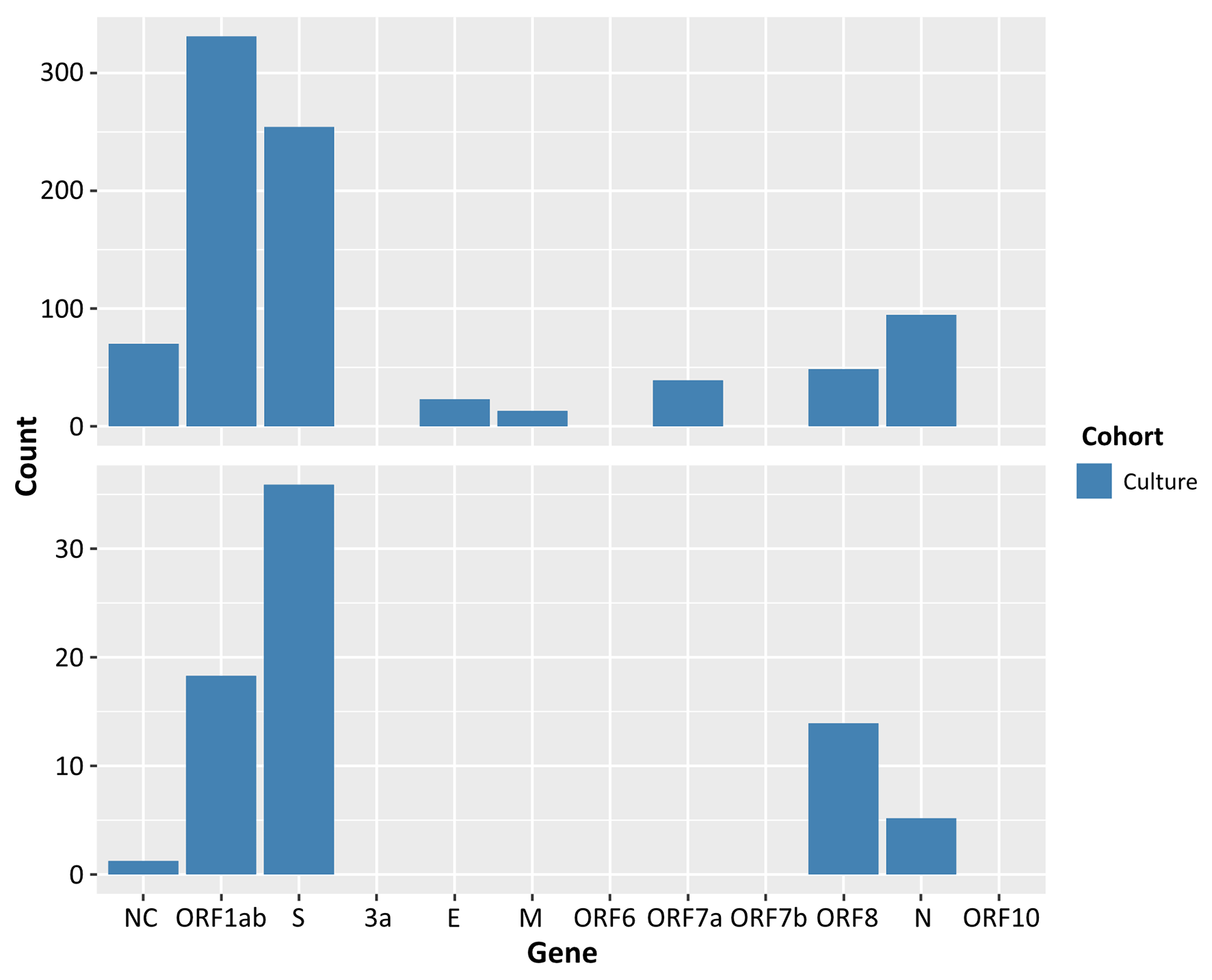

Supplement: Supplementary Figure 3 — Counts of SNPs (A) and iSNVs (B) by SARS-CoV-2 gene for the culture cohort. Frequencies ≥ 0.9 were considered SNPs. Problematic sites are not included. NC signifies non-coding region of the genome. [file Image_3.TIFF]

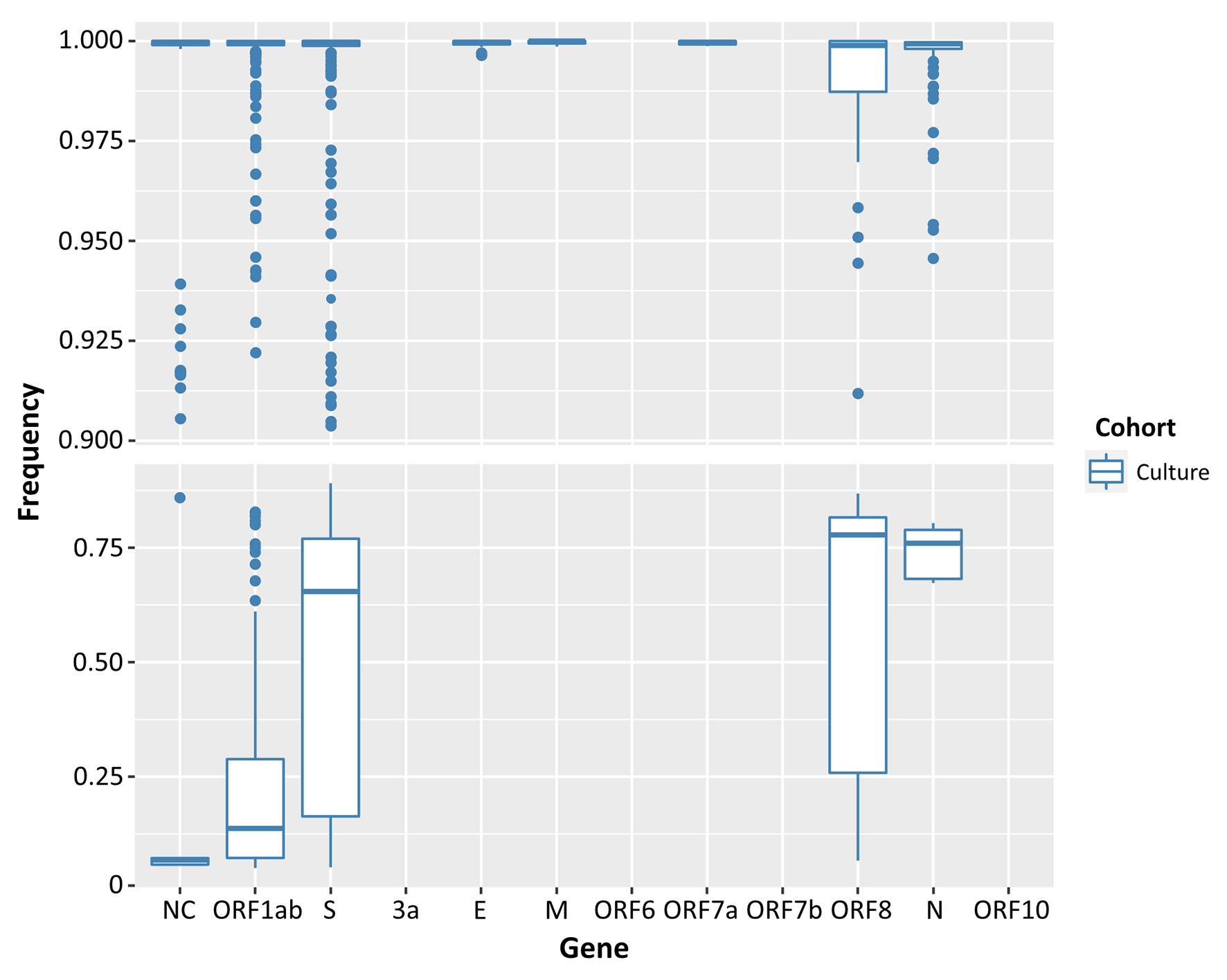

Supplement: Supplementary Figure 4 — Frequencies of SNPs (A) and iSNVs (B) by SARS-CoV-2 gene for culture cohort. Frequencies ≥ 0.9 were considered SNPs. Problematic sites are not included. NC signifies non-coding region of the genome. [file Image_4.TIFF]

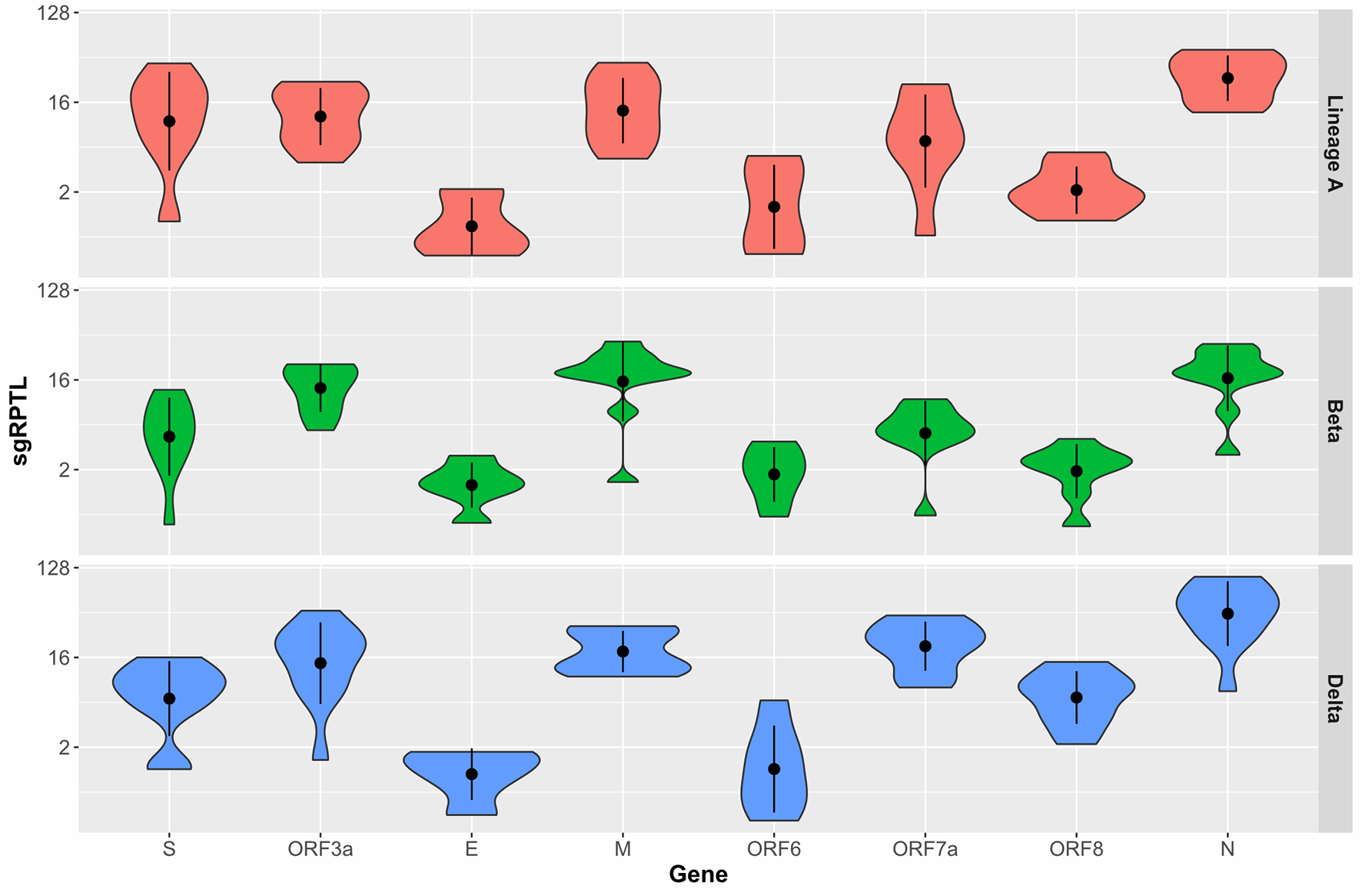

Supplement: Supplementary Figure 5 — Violin plots depicting the sgRPTL normalized counts for sgRNA abundance at each gene from top to bottom of Culture A, Culture Beta, and Culture Delta. There was significantly higher sgRNA across all genes in Delta compared to A and Beta. A and Beta were not significantly different. Individually, N was significantly higher in Delta compared to Beta and ORF 7a and 8 were significantly high than both A and Beta. The boxplots within the violins indicate the median and the interquartile ranges (25th to 75th percentile). [file Image_5.TIFF]

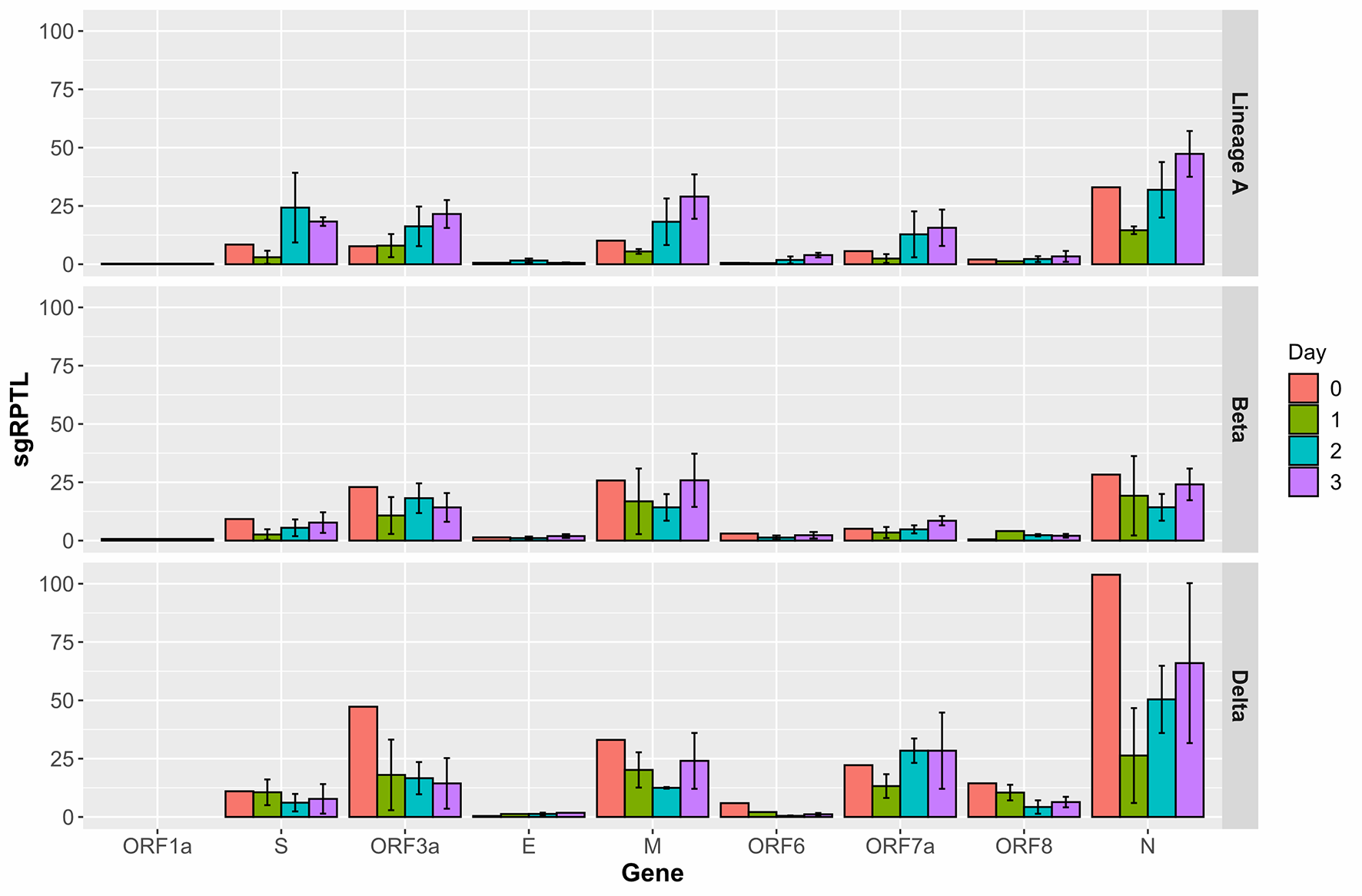

Supplement: Supplementary Figure 6 — Median sgRPTL by SARS-CoV-2 gene from inoculum (day 0) to day 3 for culture dilutions (top) lineage A, (middle) Beta, and (bottom) Delta. [file Image_6.TIFF]
